# Training of Feed-Forward Neural Networks by Using Optimization Algorithms Based on Swarm-Intelligent for Maximum Power Point Tracking

**DOI:** 10.3390/biomimetics8050402

**Published:** 2023-09-01

**Authors:** Ebubekir Kaya, Ceren Baştemur Kaya, Emre Bendeş, Sema Atasever, Başak Öztürk, Bilgin Yazlık

**Affiliations:** 1 Department of Computer Engineering, Engineering Architecture Faculty, Nevsehir Haci Bektas Veli University, Nevşehir 50300, Türkiye; emrebendes@nevsehir.edu.tr (E.B.); sema@nevsehir.edu.tr (S.A.); basakozturk@nevsehir.edu.tr (B.Ö.); bilginyazlik@nevsehir.edu.tr (B.Y.); 2 Department of Computer Technologies, Nevsehir Vocational School, Nevsehir Haci Bektas Veli University, Nevşehir 50300, Türkiye; ceren@nevsehir.edu.tr

**Keywords:** swarm intelligence, feed-forward neural network, maximum power point tracking, metaheuristic algorithm

## Abstract

One of the most used artificial intelligence techniques for maximum power point tracking is artificial neural networks. In order to achieve successful results in maximum power point tracking, the training process of artificial neural networks is important. Metaheuristic algorithms are used extensively in the literature for neural network training. An important group of metaheuristic algorithms is swarm-intelligent-based optimization algorithms. In this study, feed-forward neural network training is carried out for maximum power point tracking by using 13 swarm-intelligent-based optimization algorithms. These algorithms are artificial bee colony, butterfly optimization, cuckoo search, chicken swarm optimization, dragonfly algorithm, firefly algorithm, grasshopper optimization algorithm, krill herd algorithm, particle swarm optimization, salp swarm algorithm, selfish herd optimizer, tunicate swarm algorithm, and tuna swarm optimization. Mean squared error is used as the error metric, and the performances of the algorithms in different network structures are evaluated. Considering the results, a success ranking score is obtained for each algorithm. The three most successful algorithms in both training and testing processes are the firefly algorithm, selfish herd optimizer, and grasshopper optimization algorithm, respectively. The training error values obtained with these algorithms are 4.5 × 10^−4^, 1.6 × 10^−3^, and 2.3 × 10^−3^, respectively. The test error values are 4.6 × 10^−4^, 1.6 × 10^−3^, and 2.4 × 10^−3^, respectively. With these algorithms, effective results have been achieved in a low number of evaluations. In addition to these three algorithms, other algorithms have also achieved mostly acceptable results. This shows that the related algorithms are generally successful ANFIS training algorithms for maximum power point tracking.

## 1. Introduction

Today, investments are being made in various energy production technologies to meet the increasing demand for energy. Among these technologies, the interest and demand for renewable energy sources, which are environmentally friendly and carbon-free, is increasing day by day. Therefore, renewable energy generation is of great importance for power generation. PV panels, one of the electric power generation systems that have these characteristics, can absorb solar energy and convert it into electrical energy. PV systems have the advantage of being easy to install and using a free energy source. However, the efficiency of PV systems depends on some external factors, such as solar radiation and ambient temperature.

MPPT methods are used to optimize nonlinear power generation due to the mentioned characteristics of photovoltaic. Thus, the objective is to generate the maximum amount of electrical energy using MPPT, using a model to control a DCDC converter. An ANN is one of the most commonly used models for MPPT [1,2]. On the other hand, training the ANN model for MPPT using traditional backpropagation is not efficient [3]. To solve this problem, metaheuristic algorithms could be used.

Metaheuristic algorithms are widely used due to their global search properties that provide efficient solutions to complex problems. With their population-based search properties, these algorithms can perform a detailed search in the search space that expresses the solution to the problem without getting stuck in local minima. Bio-inspired algorithms, which mimic the behavior of intelligent swarm intelligence in nature, construct a unique interaction model between search agents. Thus, they not only perform local searches but also contribute to global searches by communicating with other agents. Therefore, they are often preferred in solving difficult optimization problems.

In this study, the MPPT of PV systems is modeled using ANN. Determining the optimal parameters of this model is a challenging optimization problem for which bioinspired swarm intelligence-based metaheuristic optimization algorithms could be used. Many variants of swarm intelligent algorithms have been proposed, especially in the last 30 years. PSO [4], ABC [5], FA [6], KHA [7], CSA [8], DA [9], GOA [10], SHO [11], BOA [12], TSA [13], TSO [14], CS [15] and SSA [16] are some of them. A total of 13 of the well-known new and old optimization algorithms from the literature were selected for this study. A comparative analysis of the algorithms’ performance in optimizing the given model was performed. The novelty of the study is that it is one of the most comprehensive studies using swarm intelligence-based algorithms for MPPT. This study makes important contributions to the literature. These contributions are presented below in substance:

- Metaheuristic algorithms are grouped according to how they occur. One of these groups is swarm intelligence-based algorithms. In this study, 13 swarm intelligence-based algorithms for FFNN training are compared. It is one of the first studies in the literature in this context.
- Metaheuristic algorithms are used to solve the MPPT problem. It is one of the most influential studies in the literature using thirteen metaheuristic algorithms for MPPT.
- The success of these algorithms in both FFNN training and MPPT will shed light on future studies.
- In this study, the effect of network structure and population size on performance is examined in detail.

The next sections of this study continue as follows: detailed related works on ANN and MPPT are given in Section 2. In Section 3, optimization algorithms based on swarm-intelligent used in this study and feed-forward neural network are introduced. Section 4 gives simulation results. The discussion is presented in Section 5. In the last section, the conclusion is given.

## 2. Related Work

Thus far, numerous methods have been suggested and put into practice for effectively supervising the MPP for PV systems. Yang et al. [17] performed four case studies, introducing the memetic SSA (MSSA), an advanced version of SSA, as a new optimization method for MPPT. It showed improved performance over other algorithms by generating more energy and reducing power fluctuations in changing weather conditions.

A Model Reference Adaptive Control (MRAC) approach was studied and compared with several established techniques, including INC, P&O, ANFIS, and Variable Step Perturb and Observe. According to the results, the MRAC approach outperformed other methods and achieved MPP in just 4 milliseconds. Its tracking ability and effectiveness were also found to be superior [18].

Kamarposhti et al. [19] employed the whale algorithm to optimize solar system parameters for enhanced MPP tracking accuracy and increased electrical power output. Their study introduced an adaptive fuzzy controller to achieve this goal. The researchers performed multiple assessments under varying irradiation conditions, comparing the performance of their proposed controller against the practical and high-performance P&O algorithm. Upon analyzing simulation outcomes, they observed that their approach outperformed the P&O algorithm.

Akram et al. [20] explored the utilization of FA for MPPT in solar PV systems. The findings demonstrated that the FA technique outperformed other methodologies, such as the P&O approach, PID control, and PSO, in relation to monitoring efficiency and convergence velocity. In contrast to PSO, which required the tuning of three parameters, the FA method only required the tuning of two parameters.

Vadivel et al. [21] focused their attention on executing the SGO algorithm. To evaluate the effectiveness of the algorithm, the researchers made a comparison with other global search MPPT techniques, including PSO, DA, and ABC. The outcomes of the simulation demonstrated the superiority of SGO-MPPT, as evidenced by the ability to achieve rapid global power tracking in under 0.2 s, coupled with a decrease in oscillations.

Conventional MPPT methods work well under stable conditions, but their effectiveness decreases during rapid changes in irradiation; this requires a fast and accurate MPPT method [22]. A few studies have compared the proposed method with conventional MPPT techniques. Examples of these studies are given below.

Mirza et al. [23] conducted a study that introduced a novel MPPT technique utilizing SSA. The SSA technique utilized the confined exploitation property of salps and improved robustness and efficiency. Also, it reduced the oscillation and saved the computation time. Comparative testing against conventional MPPT techniques showed that the SSA technique successfully handled different weather conditions and achieved faster tracking and more stable output.

Jamshidi et al. [24] presented a new technique for MPPT in solar panels utilizing a Backstepping Sliding Mode Controller (BSMC) strategy. To guarantee the stability of the proposed approach, they employed Lyapunov criteria and a fuzzy inference system. Additionally, the parameters of the Fuzzy BSMC were optimized by means of a PSO technique. The findings of the study showed that the recommended controller surpassed conventional methodologies, indicating improved performance.

Pal et al. [25] conducted a comparative analysis of their study, which put forth a novel algorithm, MPPT, in PV systems with other contemporary findings. Their outcomes exhibited a reduction in iteration and tracking time, thereby augmenting the average tracking efficiency of the proposed approach.

Mirza et al. [26] applied two new techniques for MPPT of PV systems and compared the results of their research with the results of PSO, P&O, CS, and SSA. The results obtained from their study in which they examined six distinct cases while keeping the steady-state oscillation below a certain value, and they obtained the least tracking time.

Yan et al. [27] proposed an algorithm called the Adaptive PSO Back Propagation Neural Network-Fuzzy Control (APSO-BP-FLC). The proposed algorithm consists of three stages. The simulation results showed that the proposed algorithm surpasses the performance of the APSO-BP, FLC, and P&O algorithms in terms of tracking accuracy, steady-state oscillation rate, and efficiency. Specifically, the proposed algorithm yielded an improvement compared to other algorithms.

Rezk et al. [28] introduced two optimization techniques, namely PSO and CS, in their paper. The performance of both techniques was compared to that of a conventional INR-based tracker. The findings of the study revealed that PSO and CS-based trackers showed superior performance. Moreover, the CS-based tracker outperformed the PSO-based tracker in terms of tracking time and the ability to converge to the global MPP in all investigated cases.

In their study on solar PV MPPT controller optimization using gray wolf optimizer, Aguila-Leon et al. [29] compared their proposed method with other techniques such as P&O and INC. Their results showed that the proposed controller works well under varying weather conditions and has low oscillations.

Castaño et al. [30] compared their studies, which suggested using the ABC algorithm for MPPT, with the traditional P&O method and revealed that the proposed method outperforms the proposed method. The researchers stated that the proposed algorithm has several advantages, such as high efficiency, no need for parameter knowledge, increased flexibility and simplicity, and fast-tracking power.

Al-Majidi et al. [3] proposed an optimized FFNN technique based on real data for predicting the MPP of PV arrays. The ANN model’s topology and initial weights were optimized using the PSO algorithm. The proposed method exhibited hourly average efficiencies of over 99.67% and 99.30% on sunny and cloudy days, respectively. The proposed method surpassed the conventional ANN, FLC, and P&O methods.

Corrêa et al. [31] proposed a new MPPT method for PV systems, which combines P&O with a modified version of the Fractional Open Circuit Voltage algorithm. This method enabled direct duty cycle control, reduced efficiency losses due to steady-state oscillations, and tracked the global MPP during PS. Validation via computer simulations and practical experiments showed that the proposed method outperformed commonly used MPPT algorithms.

Dagal et al. [32] investigated an enhanced version of the SSA that is based on the PSO approach. The outcomes of the study demonstrated impressive results, with the proposed algorithm achieving remarkable efficiencies of up to 99.99% and generating high power outputs of up to 316.32 W and 428.6 W under optimal operating conditions.

Ahmed et al. [33] introduced an enhanced MPPT technique that integrated the principles of the P&O method and the Fractional-Order Sliding-Mode Predictive Control (FS-MPC). This technique eliminated all the drift loops associated with traditional methods. Compared to the direct and FS-MPC methods, the proposed technique was more efficient, required fewer sensors, and had a lower computational time.

Ibrahim et al. [34] introduced a hybrid MPPT algorithm that utilized PSO for optimizing the output power of PV systems. The effectiveness of this algorithm was compared to conventional methods under various weather conditions using a grid-connected PV system. The results showed that the hybrid MPPT algorithm outperformed conventional methods with a fast-tracking time of 43.4 ms and a high efficiency of 99.07%.

Ibnelouad et al. [35] proposed a hybrid approach called ANN-PSO that utilized ANN for the prediction of solar irradiation and cell temperature, along with PSO for power generation optimization and solar power tracking. Simulation results demonstrated that the ANN-PSO approach attained up to 97% efficiency, indicating its potential to extract optimal power and enhance the performance of PV systems.

Kumar et al. [36] proposed a new hybrid algorithm that PSO-trained ML and flying squirrel search optimization to achieve optimum efficiency. The proposed algorithm is compared with other well-known methods. The findings of the study outperformed well-known algorithms, improving efficiency and reducing settling time.

Mohebbi et al. [37] introduced a novel method that integrated PSO with variable coefficients and P&O techniques. The proposed method was used to converge to the global MPP, while the P&O method was used until significant changes occurred in the system. In the study, in which the MPPT structure of the PV system is introduced under partial shading conditions, it is stated that the proposed algorithm has a much better performance than other methods, such as PSO and P&O.

Ngo et al. [38] proposed a hybrid method that combines the improved CSO and the INC algorithm for a DC standalone PV energy conversion system. The proposed method achieved the global MPP under uniform solar irradiance and PS effects, and it was faster and simpler in calculation than other methods. Simulation and experimental results demonstrated that this control strategy had been successful in searching the global region.

Nancy Mary et al. [39] presented a hybrid genetic-particle swarm-based (GA-PSO) MPPT and optimized ZETA converter to maximize output power and reduce ripple current. The GA-PSO-based ZETA converter provides regulated load power from renewable energy sources and reduces distortions in the output effectively. The total harmonic distortion (THD) was also effectively lowered by 20%.

Al-Muthanna et al. [40] discussed the challenges of using PSO for MPPT in PV energy systems, especially under PS circumstances. The paper proposes a hybrid PSO-PID algorithm that combines PSO with a PID controller to enhance tracking efficiency, reduce power ripples, and improve response time. The algorithm outperforms conventional PSO and bat algorithm MPPT strategies. The proposed hybrid PSO-PID MPPT shows significant improvement.

Kacimi et al. [22] implemented a new combined GOA-Model Predictive Controller (MPC) based GMPPT technique. Experimental simulations showed that this method outperformed traditional methods (PSO, PSO-MPC, and GOA). The integration of the MPC controller with the GOA technique reduced the steady-state oscillations and overall MPPT time.

Nisha and Nisha [41] proposed a new methodology for optimal tuning of PV systems under PS conditions by integrating hybrid MPPT algorithms. The proposed system addresses issues caused by clouds, trees, dirt, and dust and computes MPPT using an adaptive model-based approach. The hybrid optimization approach combined the CS-P&O and INC-PSO algorithms to achieve MPPT with 99.5% efficiency.

Gong et al. [42] proposed a bionic two-stage MPPT control strategy to improve the accuracy and rapidity of the MPPT controller for PV systems. The first stage used an improved ABC algorithm to quickly identify the rough search region around the global peak, while the second stage used the simultaneous heat transfer search algorithm to accurately acquire the global MPP. The proposed strategy showed excellent performance and outperformed all counterparts.

Babes et al. [43] developed an FFNN model, which is optimized with the ACO learning algorithm to evaluate the MPP of a PV system. The voltage and current of the PV array were set as the input layer of the model, and the duty cycle was set as the parameter of the output layer. Six topologies were created to find the best structure, and the best model was observed as a model with a single hidden layer and 20 neurons.

Avila et al. [44] proposed a deep reinforcement learning (DRL) algorithm to maximize the efficiency of MPPT control. With this proposed neural network, sensory information was taken as input, and the control signal was taken as output.

Saravanan et al. [45] proposed an RBFN-based MPPT algorithm, and the results were compared with the conventional P&O and INC methods using MATLAB/Simulink software. The results revealed that the RBFN algorithm is better than the conventional methods.

## 3. Materials and Methods

### 3.1. Optimization Algorithms Based on Swarm-Intelligent

#### 3.1.1. Particle Swarm Algorithm

PSO is a metaheuristic swarm intelligence-based optimization algorithm that simulates the social behavior of swarms, such as flocks of birds or schools of fish [4]. A particle in a swarm searches for food using previous experiences and discoveries of all particles in the swarm. The particles are initially randomly distributed in the search space and move according to two values related to the fitness value. The first value is the local best solution that the particle has found so far. The second value is the global best solution found by the swarm. First, each particle orders its acceleration by distance as follows:

(1) $$v_{i}=v_{i}+2r_{1}\left(x_{iBest}^{t}-x_{i}^{t}\right)+2r_{2}\left(x_{Best}^{t}-x_{i}^{t}\right)$$

Here, $r_{1}$ and $r_{2\;}$ are random values in [0, 1]. $x_{iBest}^{t}$ is the best solution of the *i*-th particle and $x_{Best}^{t}\;$ is the best solution for the swarm so far. After that, each particle moves to the next position by using the following equation.

(2) $$x_{i}^{t+1}=x_{i}^{t}+v_{i}$$

#### 3.1.2. Artificial Bee Colony Algorithm

ABC algorithm is a metaheuristic algorithm inspired by the swarming behavior of bees to find a food source [5]. Three types of artificial bees perform search operations in ABC. The first type of bee is the employed bee, which searches the food area independently. Each employed bee has a food source. Another type is onlooker bees, which select a food source from employed bees. In ABC, the colony consists of the same number of employed bees and onlooker bees, and a food source improved by only one employed bee and a random number of onlooker bees. In a predetermined iteration, an employed bee that cannot find a better food source transforms into the last type of bee, the scout bee, which performs random exploration. At the initialization of ABC, the employed bees randomly take their positions. After that, each type of bee performs exploration successively throughout the iterations. First, the employed bees search for a better position at the neighboring food source as a candidate solution as follows:

(3) $$v_{i,j}^{t}=x_{i,j}^{t}+\phi_{i,j}\left(x_{i,j}^{t}-x_{k,j}^{t}\right)$$

where jϵ{1,2,⋯D}  for *D* dimensional search space, and *k* is randomly selected bee index. *t* is the iteration index. $\phi_{i,j}\;$ is a random number in [−1, 1]. If a new location is better, the bee moves there. In the second step, onlooker bees choose a location with a probability calculated depending on the fitness values of the employed bees and behave as an employed bee. In the last step, an employed bee whose position has not changed for a certain number of iterations moves randomly in the search space. This step is the scout bee step. These three steps continue until the stop criterion is met.

#### 3.1.3. Firefly Algorithm

FA is a metaheuristic algorithm that simulates the flashing behavior of fireflies to find an optimal solution [6]. The objective function determines the brightness of a firefly with which it attracts other fireflies. The attraction also depends on the distance between the two fireflies. The greater the distance, the less the attraction. A firefly updates its position relative to the brightest firefly in proportion to the distance. If the brighter firefly is not found, it goes to a random position. The updating of the position of a firefly is formulated as follows:

(4) $$x_{i}^{t+1}=x_{i}^{t}+e^{-\lambda r^{2}}\left(x_{b}^{t}-x_{i}^{t}\right)+\alpha \varepsilon$$

where $x_{i}^{t}\;$ is the current location of the firefly and $x_{b}^{t}\;$ is the location of the brightest firefly. λ is the light absorption coefficient, and r is the distance. α is a random value in [0, 1], and ε is a random vector. The algorithm starts with randomly generated fireflies. The brightness is calculated as a function of distance and fitness value as follows:

(5) $$I\left(r\right)=I_{0}e^{-\lambda r^{2}}$$

where $I_{0}\;$ is the fitness value of the other fireflies. A firefly selects the best I(r) value as brightest and then flies toward it. If there is no brighter value, it flies randomly. These steps continue until the algorithm stops.

#### 3.1.4. Krill Herd Algorithm

KHA is a metaheuristic algorithm that simulates the density-dependent attraction and foraging of krill populations [7]. Three operations determine the movement of a krill in search space. The first is the movement influenced by other krill individuals and is calculated as follows:

(6) $$N_{i}^{t+1}=N^{max}\alpha_{i}+\omega_{i}N_{i}^{t}$$

Here, $\alpha_{i}=\alpha_{i}^{local}+\alpha_{i}^{target}$. $\alpha_{i}^{local}\;$ is the local effect caused by the neighbor and $\alpha_{i}^{target}\;$ is the direction determined by the best krill individual. $N^{max}\;$ is the maximum induced speed, $\omega_{i}\;$ is the inertia weight of induced motion in [0, 1], and $N_{i}^{t}\;$ is the last induced motion. The second operation is the foraging motion, which is calculated based on the food location and previous experience. The foraging motion is expressed as follows:

(7) $$F_{i}^{t+1}=V_{f}\beta_{i}+\omega_{f}F_{i}^{t}$$

where $\beta_{i}=\beta_{i}^{food}+\beta_{i}^{best}$ which $\beta_{i}^{food}\;$ is the attractiveness of the food and $\beta_{i}^{best}\;$ is the effect of best fitness. $V_{f}\;$is the speed of foraging and $\omega_{f}\;$ is the inertia weight for foraging motion in [0, 1]. The krill individuals move randomly in the search space at the last operation, which is called physical diffusion, as follows:

(8) $$D_{i}^{t+1}=D^{max}\delta$$

Here, $D^{max}\;$ is the maximum diffusion speed, and δ is a random vector. Using all three motion operations above, the final motion of the krill is calculated as follows:

(9) $$x_{i}^{t+\Delta t}=x_{i}^{t}+\Delta t\left(N_{i}^{t+1}+F_{i}^{t+1}+D_{i}^{t+1}\right)\;\Delta t=C_{t}\overset{n}{\underset{j=1}{\sum }}\left(U_{j}-L_{j}\right)$$

where *n* is the dimension of the search space, $U_{j}$ is the upper bound and $L_{j}\;$ is the lower bound of the *j*th dimension. $C_{t}\;$ is a constant in [0, 2]. In the last step, for the sake of improving the performance of the algorithm, genetic operators such as the crossover and the mutation are implemented. All these steps form a KHA iteration that continues until stopping conditions are met.

#### 3.1.5. Chicken Swarm Optimization

CSO is a bio-inspired metaheuristic optimization algorithm that mimics the behaviors of a chicken swarm [8]. The swarm is divided into groups consisting of one roaster, a number of hens, and chicks. Chickens are ranked according to their fitness values. The best *RN* individuals are assigned as roosters, the worst *CN* individual chicks, and the rest as hens. The roster whose fitness value is better has a bigger probability of searching a wider range of areas. The new position of a roster is calculated as follows:

(10) $$x_{i}^{t+1}=x_{i}^{t}\left(1+rand\left(0,\sigma^{2}\right)\right)$$

Here, $rand\left(0,\sigma^{2}\right)\;$ is a Gaussian distribution in which the mean is 0 and standard deviation is $\sigma^{2}$. A, hence, is moved by following the roster in the same group, and this is formulated as follows:

(11) $$x_{i}^{t+1}=x_{i}^{t}+re^{\left(\frac{f_{i}-f_{r1}}{\left|f_{i}\right|+\varepsilon }\right)}\left(x_{r1}^{t}-x_{i}^{t}\right)+re^{\left(f_{r2}-f_{i}\right)}\left(x_{r2}^{t}-x_{i}^{t}\right)$$

where *r* is a random value in [0, 1]. $x_{r1}^{t}\;$ is the position of the roster while $x_{r2}^{t}\;$ is a position of any chicken in the group. The *f* is the fitness value of a chicken, and ε is smallest value a computer can produce. The chicks search around their mother with the following formula.

(12) $$x_{i}^{t+1}=x_{i}^{t}+F\left(x_{m}^{t}-x_{i}^{t}\right)$$

where *F* is a constant in [0, 2] and $x_{m}^{t}\;$ is the position of the chick’s mother. After *G* iterations, the status of chickens is rearranged, and each chicken moves depending on its status at each iteration until CA stops because of stopping conditions.

#### 3.1.6. The Dragonfly Algorithm

DA is one of the swarm intelligent optimization algorithms whose main inspiration is the swarm behavior of dragonflies during hunting and migration [9]. Individuals in the swarm change their position based on five factors: separation, alignment, cohesion, attraction, and distraction. Separation is expressed by the sum of the distances of an individual location from other individual locations. Alignment is calculated by taking the average of neighboring locations, and cohesion is the difference between the alignment and the current individual location. Attraction is the distance to the food source, and distraction is the sum of the enemy and current individual positions. The food source is the best solution, while the enemy is the worst solution ever. The position is updated using the following formula.

(13) $$x_{i}^{t+1}=x_{i}^{t}+\Delta x_{i}^{t+1}$$

where $x_{i}^{t}\;$ current position of *i*th individual, *t* is iteration counter and $\Delta x_{i}^{t+1}\;$ is step vector formulated as follows:

(14) $$\Delta x_{i}^{t+1}=\left(sS_{i}+aA_{i}+cC_{i}+fF_{i}+eE_{i}\right)+w\Delta x_{i}^{t}$$

where *s*, *a*, *c*, *f,* and *e* are, respectively, the separation coefficient, the alignment coefficient, the cohesion coefficient, the attraction coefficient, and the distraction coefficient. *w* is inertia weight. $S_{i}$, $A_{i}$, $C_{i}$, $F_{i}$ end $E_{i}$ are the separation, alignment, cohesion, attraction, and distraction values, respectively. A dragonfly has a neighborhood within a certain radius. During iterations, the radius is increased to explore a global optimum. If there is no other dragonfly in the neighborhood, the dragonfly uses a random walk as follows:

(15) $$x_{i}^{t+1}=x_{i}^{t}+L\mathrm{evy}\left(d\right)\times x_{i}^{t}$$

where *d* is the dimension of the search space and *levy(.)* is the Levy flight function. Low cohesion and high alignment weights are used with a small radius. During iteration, the larger the radius, the smaller the alignment, the greater the cohesion. Accordingly, at each iteration, parameters should be updated until the stopping criterion is met.

#### 3.1.7. Grasshopper Optimization Algorithm

GOA is an optimization algorithm that simulates the behavior of grasshopper swarms [10]. First, the parameters of GOA, such as maximum iteration, population size, *cmax,* and *cmin* are defined, and the fitness of each grasshopper is calculated. At each iteration, the c coefficient is updated as follows:

(16) $$c=cmax-l\frac{cmax-cmin}{L}$$

where *l* is the current iteration, and *L* is the maximum iteration. *cmax* and *cmin* are the maximum and minimum values for *c*, respectively. After implementing this formula, for each grasshopper, the distances between grasshoppers are normalized into [1, 4], and the new positions are calculated as follows:

(17) $$x_{i,d}^{t+1}=c\left(\overset{N}{\underset{\begin{array}{c} j=1\\ j\neq i \end{array}}{\sum }}c\frac{ub_{d}-lb_{d}}{2}\left(fe^{\frac{-\left|x_{j,d}^{t}-x_{i,d}^{t}\right|}{l}}-e^{-\left|x_{j,d}^{t}-x_{i,d}^{t}\right|}\right)\frac{x_{j}^{t}-x_{i}^{t}}{\left|x_{j}^{t}-x_{i}^{t}\right|}\right)+\hat{T_{d}}$$

Here, N is the total number of grasshoppers in the swarm, *f* is the intensity of attraction, *l* is the attractive length scale. $ub_{d}\;$ and $lb_{d}\;$ are the upper and lower bounds of the *d*th dimension of the search space. $\hat{T_{d}}\;$ is the best position till now. The iterations continue until the maximum iteration is reached, and finally, the best position is returned as the solution of GHA.

#### 3.1.8. Selfish Herd Optimizer

SHO is a swarm optimization algorithm [11]. The population consists of two groups: Pack predators (P) and Perd of Prey (H). Each member of the population has a survival value computed by the following equation.

(18) $$SV_{i}=\frac{f_{i}-f_{best}}{f_{best}-f_{worst}}$$

where $f_{i}\;$ is the fitness value of the position of the *i*th member. $f_{best}$ and $f_{worst}$ are the best and worst fitness values found so far by SHO. Through the SHO iterations, the herd movement operator is first applied for each member in H, which consists of two types of movements: herd’s leader movement and herd’s following and desertion movement. The position of the leader of a herd, which is the best in H so far, is updated as follows:

(19) $$x_{L}^{t+1}=\{\begin{array}{c} x_{L}^{t}+c^{t}\;\;if\;SV_{L}=1\\ x_{L}^{t}+s^{t}\;\;if\;SV_{L}<1 \end{array}$$

where $c^{t}\;$ is the motion vector that depends on the selfish repulsion experiment and $s^{t}$ is the motion vector that depends on the selfish attraction experiment. Within H, the members except the leader are divided into groups of herd followers (H_F_) and herd deserters (H_D_), and each member moves according to the equation below.

(20) $$x_{i}^{t+1}=\{\begin{array}{c} x_{i}^{t}+f_{i}^{t}\;\;if\;i\in H_{F}^{t}\\ x_{i}^{t}+d_{i}^{t}\;\;if\;i\in H_{D}^{t} \end{array}$$

Here $f_{i}^{t}\;$ is the following movement vector and $d_{i}^{t}\;$ is the herd desertion vector in the iteration *t*. Then, the predator movement operator is applied to each member in P.

(21) $$x_{i}^{t+1}=x_{i}^{t}+2\rho \left(x_{h}^{t}-x_{i}^{t}\right)$$

Here $x_{h}^{t}\;$ is the position of a randomly selected member in H, ρ is a random number in [0, 1]. The iteration of SHO ends after predation and restoration phases are performed. In the predation phase, members of the herd are killed by predators with the probability of being hunted. These members are removed from the population. In the restoration phase, new herd members are generated from the remaining herd members with the mating probability. After these phases, if the stop criterion is satisfied, the iteration is finished.

#### 3.1.9. The Butterfly Optimization Algorithm

BOA is inspired by the food-foraging activities of butterflies [12]. The optimization process consists of three phases: The initial phase, the iteration phase, and the final phase. Each phase is executed sequentially. In the initial phase, randomly generated agents, called butterflies, are created to represent a candidate solution. Also, in this phase, the solution space and the fitness function are defined, and the algorithm parameters are initialized. The butterflies use the fragrance of other butterflies to find nectar, and each butterfly generates the fragrance according to its fitness value. The fragrance is calculated as follows:

(22) $$f=cI^{a}$$

where *c* and *a* are the sensory modality and power exponent, respectively, and are in the range [0, 1]. *I* is the fitness value of the solution and is referred to as the stimulus intensity. In the second phase, iterations are performed until the stopping criteria are met. A new position of each butterfly is computed by one of the two main operations: global search and local search. A switch probability *p* determines whether the global search or the local search is used. In global search, the direction of movement to find the new position is determined by using the best position g* as follows:

(23) $$x_{i}^{t+1}=x_{i}^{t}+\left(r^{2}\times g^{*}-x_{i}^{t}\right)\times f_{i}$$

where $x_{i}^{t}\;$ is the solution of the *i*th butterfly and *r* is a random number in [0, 1]. If *r* is greater than the switch probability, then the local search is performed instead, as follows:

(24) $$x_{i}^{t+1}=x_{i}^{t}+\left(r^{2}\times x_{j}^{t}-x_{k}^{t}\right)\times f_{i}$$

where $x_{j}^{t}\;$ and d $x_{k}^{t}$ are the solution of the *i*th and *j*th butterflies. The final phase of BOA indicates the achievement of the stopping criterion. In this phase, the best butterfly position is determined as the solution.

#### 3.1.10. Tunicate Swarm Algorithm

TSA is a bio-inspired metaheuristic optimization algorithm [13]. Jet propulsion and swarm intelligence are two behaviors of tunicates used by TSA. Jet propulsion is modeled by three conditions. Avoiding conflicts between search agents, the first condition uses a vector to determine the new position of a tunicate as follows:

(25) $$\vec{A}=\frac{c_{2}+c_{3}-2c_{1}}{\left\lfloor P_{min}+c_{1}P_{max}-P_{min}\right\rfloor }$$

Here $c_{1}$, $c_{2}$, and $c_{3}$ are random numbers in [0, 1]. $P_{min}$ and $P_{max}$ give the speed limits for social interaction. The second condition is the movement towards the best neighbor. For this purpose, the distance between the tunicate and the food source is calculated as follows:

(26) $$\vec{PD}=\left|\vec{FS}-r\vec{P_{p}\left(x\right)}\right|$$

where $\vec{FS}$ represents the optimal location, *x* indicates the current iteration, and $\vec{P_{p}\left(x\right)}$ indicates the position of the agent. *r* is a random value in [0, 1]. Then, the last condition for modeling the jet propulsion is performed by converging to the best search agent as follows:

(27) $$\vec{P_{p}\left(x\right)}=\{\begin{array}{c} \vec{FS}+\vec{A}\cdot \vec{PD}\;\;\;\;if\;r\geq 0.5\\ \vec{FS}-\vec{A}\cdot \vec{PD}\;\;\;\;if\;r<0.5 \end{array}$$

When modeling the swarm behavior of tunicates, the best solution is used to update the positions of the agents in the swarm using the following equation.

(28) $$\vec{P_{p}\left(x+1\right)}=\frac{\vec{P_{p}\left(x\right)}+\vec{P_{p}\left(x+1\right)}}{2c_{1}}$$

At each iteration, the TSA algorithm updates the search accents and their position with respect to the jet propulsion and swarm behavior and returns the best optimal position so far as the solution.

#### 3.1.11. Tuna Swarm Optimization

TSO is a swarm-based metaheuristic optimization algorithm based on the cooperative foraging behavior of tuna schools [14]. The spiral and parabolic tuna foraging strategies are adapted to the TSO algorithm. Which strategy is used in an iteration is determined randomly. In the spiral strategy, the next position of the tunas is calculated as follows:

(29) $$x_{i}^{t+1}=\left(\alpha +\left(1-\alpha \right)t/t_{max}\right)\left(x_{best}^{t}+\beta \left|x_{best}^{t}-x_{i}^{t}\right|\right)+\left(\left(1-\alpha \right)-\left(1-\alpha \right)t/t_{max}\right)x_{i-1}^{t}$$

where $\beta =e^{bl}\cos\left(2\pi b\right)\;$ and $l=e^{3\cos\left(\left(\left(t_{max}+1/t\right)-1\right)\pi \right)}$. *a* is a constant, *t* is the number of iterations, $t_{max}$ is the maximum number of iterations, i is tuna, and b is a random number in [0, 1]. To improve global exploration capability, a randomly generated position is swapped with $x_{best}^{t}$ in the equation mentioned above, provided that a randomly generated value from the interval [0, 1] is greater than the ratio of $t/t_{max}$. In the case of using parabolic foraging to determine the next position in an iteration, the equation given below is used.

(30) $$x_{i}^{t+1}=\{\begin{array}{cc} x_{best}^{t}+r\left(x_{best}^{t}-x_{i}^{t}\right)+TF{\left(1-t/t_{max}\right)}^{2t/t_{max}}\left(x_{best}^{t}-x_{i}^{t}\right) & if\;r<0.5\\ TF{\left(1-t/t_{max}\right)}^{2t/t_{max}}x_{i}^{t} & if\;r\geq 0.5 \end{array}$$

where TF is a random value in [−1, 1]. The TSO iterations continue to search for optimum results until the stopping criteria are met.

#### 3.1.12. Cuckoo Search

CS is a metaheuristic optimization algorithm inspired by the obligate brood parasitic behavior of some cuckoo species [15]. An egg is a solution produced by a cuckoo and stored in a nest in CS. The initial population is created by randomly generated eggs for each host nest. Only one cuckoo is placed in a nest, and a new solution is generated from a randomly selected egg by the cuckoo as follows:

(31) $$x_{i}^{t+1}=x_{i}^{t}+\alpha \times L\mathrm{evy}\left(\lambda \right)$$

Here, α is the step size. In most cases α = 1 and 1<λ≤3. Each cuckoo places its egg in a randomly selected nest at a time if the new egg is better than the older one. The best nests, containing high-quality eggs, are retained to pass on to the next generation, while the $p_{a}$ potion of the remainder is reproduced. These steps are repeated until the stopping criterion is met.

#### 3.1.13. Salp Swarm Algorithm

SSA was proposed to solve engineering optimization problems using the navigation and foraging behavior of salps as a model [16]. The salp that has the best position is called the leading salp. The other members of the population are called follower salps, which form a salp chain to chase the position of the leading salp. First, an SSA population is randomly created, and the leading salp is determined. Then, the position of the leading salp is updated as follows:

(32) $$x_{1}^{t+1}=\{\begin{array}{cc} x_{1}^{t}+c_{1}\left(\left(u-l\right)c_{2}+l\right) & c_{3}\geq 0\\ x_{1}^{t}-c_{1}\left(\left(u-l\right)c_{2}+l\right) & c_{3}<0 \end{array}$$

where $x_{1}^{t}$ is the position of the leading salp at the *t*-th iteration. u and l are the upper and lower bounds of the search space, respectively. $c_{2}$ and $c_{3}$ are random numbers in [0, 1]. The value of $c_{1}$ is updated at each iteration as follows:

(33) $$c_{1}=2e^{-{\left(\frac{4t}{t_{max}}\right)}^{2}}$$

Here, $t_{max}$ is the maximum number of iterations, and t is the current iteration. After the position of the leader has been determined, the position of the followers is determined as follows:

(34) $$x_{i}^{t+1}=1/2\left(x_{i}^{t}+x_{i-1}^{t}\right)$$

Here, $x_{i}^{t}$ is the *i*-th position of the follower salp *i* ≥ 2. Until reaching the global optimum or stopping criteria, these processes are repeated.

### 3.2. Feed Forward Neural Network

While developing ANNs, as the name suggests, it was inspired by the nervous system of humans. ANN is successfully used to solve many problems, such as pattern classification, signal processing, image processing, and prediction. The most important feature that distinguishes ANNs from classical computer programs is their ability to learn. ANNs, or in short, neural networks, are classified under two main headings: FFNNs and RNNs. The main difference between the two models is that there is no feedback in FFNNs [46]. FFNNs can approximate any function assigned to it with targeted accuracy [47].

There are no closed paths in FFNN models. Input and output nodes are not interconnected among themselves; all other nodes are hidden nodes. Once the input nodes are set, the remaining points adjust their values by forward propagation. In FFNN, the output node values are determined by optimizing the model according to the values given at the input [48].

**Figure 1:**
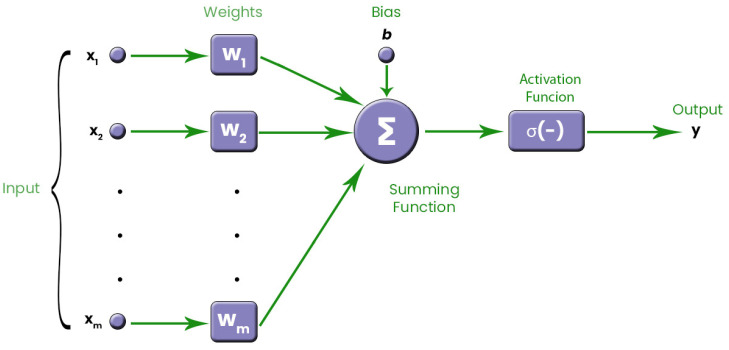
Conventional model of an artificial neuron.

Artificial neurons are the heart of ANNs. We use many artificial neurons to form an ANN. In a conventional FFNN, there are three layers: the input layer, the hidden layer, and the output layer. As shown in Figure 1, a neuron produces an output by performing a series of operations on the data applied to its input. A FFNN can be formed by connecting the output of one neuron to the input of another neuron. The neuron model in Figure 1 has many inputs but only one output. Activation and transfer functions have a key role in the output [49].

Input values are weighted using *w* values. The bias value is denoted by *b*. The activation function is denoted by *f*. The output of the artificial neuron is denoted by *y*. There are different activation functions used in ANNs. One of the commonly used functions among these functions is the sigmoid function. In this study, we use the sigmoid function. The calculations performed in an artificial neuron are as follows:

(35) $$y=f\left(\overset{m}{\underset{i=1}{\sum }}w_{i}x_{i}+b\right)$$

(36) $$\sigma \left(x\right)=\frac{1}{1+e^{-x}}$$

Weights and bias values have a significant effect on the training of the network. The total number of parameters to be optimized depends on the number of inputs, the total number of neurons, and their weights.

## 4. Simulation Results

In this study, FFNN was trained by using swarm-intelligent-based metaheuristic algorithms for MPPT, and their performances were analyzed. 13 algorithms were used for FFNN training. These algorithms are ABC, BOA, CS, CSO, DA, FA, GOA, KHA, PSO, SHO, SSA, TSA, and TSO.

**Figure 2:**
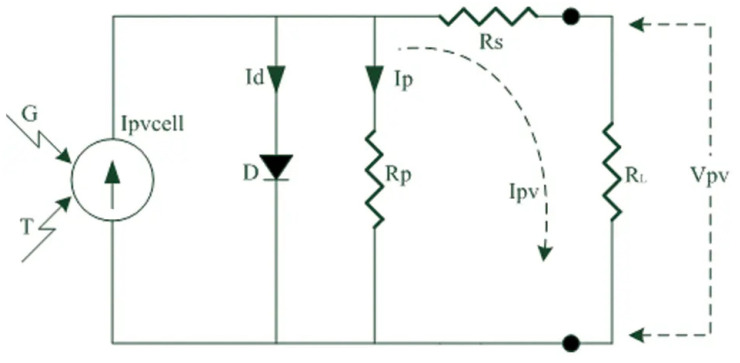
The simplified electrical equivalent circuit of a PV cell [50].

One of the most important techniques used to reach the maximum power of alternative energy sources is MPPT. Although it is used in different energy sources, its most intensive use is on wind turbines and solar panels [50]. This study is on the MPPT of the solar PV system. The simplified electrical equivalent circuit of a PV cell is given in Figure 2. It is possible to calculate the output current and output voltage of a PV cell via (37) and (38). Here, pvcell is PV cell current depending on temperature and solar radiation. Id is diode current. Ip is parallel resistor current. Vd is diode voltage drop. Ipv is the PV cell output current. Rs is serial resistor [50]. In this study, the data of 250 W solar panels was used. The inputs of the system are temperature (t) and solar radiation (s). The power value (p) is the output of the system. As here, the data set is scaled in the range of [0, 1] due to the large values. All analyses were performed according to the scaled data set.

(37) Ipv=Ipvcell−Id−Ip

(38) Vpv=Vd−Ip∗Rs

**Figure 3:**
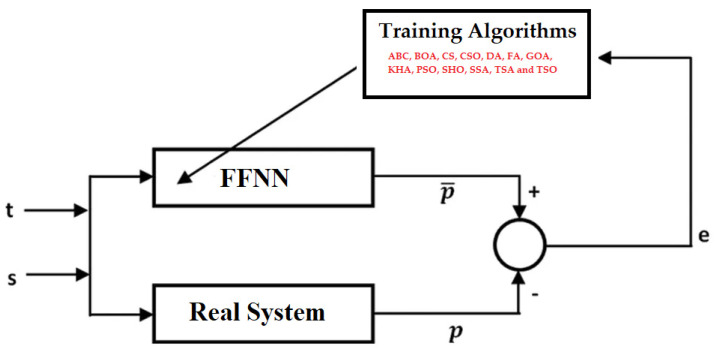
Block diagram of FFNN-based MPPT modeling.

Approximately 80% of the data set was used for the training process. The rest is devoted to the testing process. The block diagram of the training process to be carried out for MPPT is presented in Figure 3. As seen here, a training process is separately carried out with ABC, BOA, CS, CSO, DA, FA, GOA, KHA, PSO, SHO, SSA, TSA, and TSO algorithms. The output p is obtained corresponding to the inputs t and s. The difference between the estimated output and the real output gives the error. It is aimed to minimize this error at the end of the training process. A zero error indicates that the real system is optimally modeled.

Each application was run at least 30 times in order to obtain statistical results. The mean squared error was used as the error metric. In the training process, results for three different population sizes were obtained for each algorithm. These values are 10, 20, and 50. The numbers of maximum generation corresponding to these population sizes are 100, 50, and 20, respectively. Other control parameters used for algorithms are as follows. The “limit” value of the ABC algorithm is calculated as (NP×D)/2. NP is the number of population size. D is the number of parameters of the problem to be optimized. The probability switch, power exponent, and sensory modality values of BOA are 0.8, 0.1, and 0.01, respectively. The discovery rate of alien eggs/solutions of CS is 0.25. The light absorption coefficient, attraction coefficient base value, mutation coefficient, and mutation coefficient damping ratio of FA are 1, 2, 0.2, and 0.98, respectively. The inertia weights of PSO are 0.9 and 0.6. The constant (a) of TSO is 0.7.

The FFNN model, consisting of two inputs and one output, was created to solve the related problem. The results obtained for models with 5, 10, and 15 neurons in the hidden layer are examined. In other words, 2-5-1, 2-10-1, and 2-15-1 network structures were used in the applications. Only three different network structures were taken into consideration, especially due to the high number of algorithms, the fact that each application was run 30 times, and results in different population sizes were obtained. In other words, high processing times are avoided. This is one of the limitations of this study. These results in this study should be considered within limitations. The summing function is used as the transfer function in FFNN. Sigmoid was chosen as the activation function. The optimized parameter numbers for 2-5-1, 2-10-1, and 2-15-1 network structures are 21, 41, and 61, respectively.

The results obtained with 13 metaheuristic algorithms for *n* = 10 are given in Table 1. The most effective training and test error values of the ABC algorithm were found to be 2-5-1. The training and test error values obtained with the ABC algorithm are 5.0 × 10^−3^ and 5.1 × 10^−3^, respectively. The worst training and test results were achieved with the BOA. Increasing the number of neurons in CS worsened the results. The best results in both training and testing were obtained with the 2-5-1 network structure. These results are 3.9 × 10^−3^ and 3.9 × 10^−3^, respectively. The best results in CSO were obtained with the 2-10-1 network structure. The training error value of CSO is 3.7 × 10^−3^. The test error value found is 3.8 × 10^−3^. Increasing the number of neurons in DA worsened the performance. The best training and test error values were obtained in the 2-5-1 network structure. Effective results were achieved in all network structures in FA. The best training and test error values were found as 1.8 × 10^−3^ and 1.8 × 10^−3^, respectively, via a 2-10-1 network structure. Increasing the number of neurons in GOA improved performance. The best training and test error values were obtained with 2-15-1. As in the GOA, the best results were achieved with 2-15-1 in KHA. The training result of KHA is 5.5 × 10^−3^. The test is 5.6 × 10^−3^. Network structure with low neuron count was more effective in PSO. Better results were found with 2-5-1. Effective results were achieved with all network structures in SHO. The best training and test results are 3.5 × 10^−3^ and 3.6 × 10^−3^, respectively. The 2-15-1 network structure has been effective in SSA. The best training error value of SSA is 4.0 × 10^−3^. The test error value is 4.1 × 10^−3^. The most effective network structure in TSA is 2-10-1. The training and test error values are 2.5 × 10^−3^ and 2.6 × 10^−3^, respectively. As in TSA, the 2-10-1 network structure is more effective in TSO.

The results obtained with 13 metaheuristic algorithms for *n* = 20 are given in Table 2. 2-5-1 is the most effective network structure in the ABC algorithm. As with *n* = 10, the results of BOA are ineffective at *n* = 20. 2-5-1 is more effective in CS. The training and test results of CS are 5.5 × 10^−3^ and 5.6 × 10^−3^, respectively. The training error value found in the CSO is 3.9 × 10^−3^. The test error value is 4.0 × 10^−3^. An increase in the number of neurons in DA improved performance. The most effective results were achieved with the 2-5-1 network structure in FA. The training and test results of the FA are 5.0 × 10^−4^ and 5.2 × 10^−4^, respectively. Effective and similar results were achieved with all network structures in GOA. The increasing population size in KHA worsened performance. Unsuccessful results were found with all network structures. The most effective results in PSO were achieved with 2-10-1. For *n* = 20, SHO is successful. Increasing the number of neurons in SHO improved performance. Effective results were achieved with 2-15-1 in SHO. The training and test error values found are 1.6 × 10^−3^ and 1.6 × 10^−3^, respectively. In SSA, 2-15-1 is more effective than other network structures. Effective results were achieved with 2-10-1 in TSA. Training and test results of TSA are 2.5 × 10^−3^ and 2.5 × 10^−3^, respectively. 2-10-1 and 2-15-1 network structures are successful in TSO.

The results obtained with 13 metaheuristic algorithms for *n* = 50 are given in Table 3. The results obtained for *n* = 50 in the ABC algorithm are unsuccessful. The training and test error values found in the 2-10-1 network structure with BOA are 1.5 × 10^−2^ and 1.5 × 10^−2^, respectively. In CS and CSO, 2-10-1 network structures are more effective than other network structures. The training error value found with the 2-5-1 network structure in DA is 3.8 × 10^−3^. Its test error value is 3.9 × 10^−3^. Effective results have been achieved with all network structures in FA. The most effective network structure is 2-5-1. The training and test error values found are 4.5 × 10^−4^ and 4.6 × 10^−4^, respectively. Successful training and test results were achieved with 2-10-1 in GOA. The results found for *n* = 50 in KHA and PSO are unsuccessful. Successful results were achieved in SHO. The 2-10-1 network structure is effective. The best training and test error values found are 1.9 × 10^−3^ and 2.0 × 10^−3^, respectively. The training and test error values found with 2-10-1 in the SSA are 5.3 × 10^−3^ and 5.4 × 10^−3^, respectively. Effective results were found with the 2-15-1 network structure in TSA. The training and test error values found with the 2-5-1 network structure in TSO are 4.1 × 10^−3^.

## 5. Discussion

Network structure and population size significantly affect the performance of 13 algorithms. Some algorithms are more effective at low population sizes, while others are more effective at higher population sizes. The same is true for the network structure. The change in the number of neurons in the hidden layer affects the performance. Information on the best train mean error values obtained using related metaheuristic algorithms is given in Table 4. The best results with ABC, CS, CSO, KHA, PSO, SSA, TSA, and TSO algorithms were obtained when *n* = 10. The most effective results with DA and SHO were found when *n* = 20. BOA, FA, and GOA achieved more effective results with *n* = 50. In other words, the most successful results in 8 of 13 algorithms were obtained when *n* = 10. This number was 2 for *n* = 20, while this value was 3 for *n* = 50. The most effective results with ABC, CS, FA, and PSO were obtained with the 2-5-1 network structure. On the other hand, BOA, CSO, GOA, TSA, and TSO achieved better results in the 2-10-1 network structure. Other algorithms found their best results with a 2-15-1 network structure. In other words, the most effective results of the five algorithms were achieved with the 2-10-1 network structure. For other network structures, this value is four. Information on the best test mean error values obtained using related metaheuristic algorithms is given in Table 5. All of the evaluations made for Table 4 are valid for Table 5. In other words, the parameters in the training and testing processes showed parallelism with each other.

According to the results in Table 4 and Table 5, the success rankings of 13 swarm intelligent-based training algorithms in the training and testing process were compared for the solution of the MPPT problem in Table 6. The most effective result was found with FA in both training and testing processes. The training and test error values obtained are 4.5 × 10^−4^ and 4.6 × 10^−4^, respectively. After FA, the most successful algorithm is SHO. The best training and testing values found with SHO are 1.6 × 10^−3^. The third most successful algorithm is GOA. The best training error achieved with GOA is 2.3 × 10^−3^. The test error value of GOA is 2.4 × 10^−3^. The fourth successful algorithm is TSA. The fifth algorithm is TSO. The next ranks according to performance are as follows: DA, CSO, CS, SSA, ABC, KHA, PSO, and BOA.

## 6. Conclusions

In this study, the performance of thirteen swarms of intelligence-based algorithms in FFNN training for MPPT was evaluated. These algorithms are ABC, BOA, CS, CSO, DA, FA, GOA, KHA, PSO, SHO, SSA, TSA, and TSO. Three different network structures are used to analyze the effect of the network structure of the ANN on the results. They are 2-5-1, 2-10-1 and 2-15-1. At the same time, the effect of population size, which is one of the important control parameters, on the results was investigated. The results are obtained for values of 10, 20 and 50. The general results of this study are as follows:

- In general, all algorithms were found to be effective for MPPT. The three most effective algorithms are FA, SHO, and GOA.
- Network structure affects the performance of training algorithms. The network structure in which each algorithm is more successful may be different from each other.
- As with the network structure, the population size affects the performance of the training algorithms in solving the related problem. The population size in which each algorithm is more successful may differ.
- In general, the training and test results for each algorithm were close to each other. This shows that the learning process is successful.

This study is one of the most comprehensive studies based on swarm intelligence, and the performance of thirteen algorithms is compared specifically to MPPT. However, there are many problems in the real world, and the results of this study will shed light on future studies. We will evaluate the performances of metaheuristic algorithms based on swarm intelligence in the future in solving problems in many fields, such as engineering, economics, social sciences, educational sciences, and medicine.

## Figures and Tables

**Table 1:** Comparison of the results obtained for *n* = 10.

System	Network Structure	The Results			
		Train		Test	
		Mean	Std.	Mean	Std.
ABC	2-5-1	5.0 × 10^−3^	2.4 × 10^−3^	5.1 × 10^−3^	2.4 × 10^−3^
	2-10-1	1.2 × 10^−2^	4.3 × 10^−2^	1.8 × 10^−2^	7.6 × 10^−2^
	2-15-1	6.0 × 10^−3^	3.2 × 10^−3^	6.0 × 10^−3^	3.1 × 10^−3^
BOA	2-5-1	5.2 × 10^−2^	4.1 × 10^−2^	5.2 × 10^−2^	4.1 × 10^−2^
	2-10-1	3.8 × 10^−2^	3.5 × 10^−2^	3.8 × 10^−2^	3.5 × 10^−2^
	2-15-1	4.1 × 10^−2^	3.8 × 10^−2^	4.1 × 10^−2^	3.8 × 10^−2^
CS	2-5-1	3.9 × 10^−3^	1.1 × 10^−3^	3.9 × 10^−3^	1.1 × 10^−3^
	2-10-1	4.0 × 10^−3^	1.2 × 10^−3^	4.1 × 10^−3^	1.2 × 10^−3^
	2-15-1	4.3 × 10^−3^	1.6 × 10^−3^	4.4 × 10^−3^	1.6 × 10^−3^
CSO	2-5-1	5.3 × 10^−3^	2.9 × 10^−3^	5.3 × 10^−3^	2.9 × 10^−3^
	2-10-1	3.7 × 10^−3^	2.6 × 10^−3^	3.8 × 10^−3^	2.6 × 10^−3^
	2-15-1	4.6 × 10^−3^	2.6 × 10^−3^	4.7 × 10^−3^	2.6 × 10^−3^
DA	2-5-1	5.0 × 10^−3^	2.9 × 10^−3^	5.0 × 10^−3^	2.9 × 10^−3^
	2-10-1	5.3 × 10^−3^	3.4 × 10^−3^	5.4 × 10^−3^	3.5 × 10^−3^
	2-15-1	5.7 × 10^−3^	3.5 × 10^−3^	5.7 × 10^−3^	3.4 × 10^−3^
FA	2-5-1	2.0 × 10^−3^	2.0 × 10^−3^	2.0 × 10^−3^	2.0 × 10^−3^
	2-10-1	1.8 × 10^−3^	1.9 × 10^−3^	1.8 × 10^−3^	1.9 × 10^−3^
	2-15-1	1.9 × 10^−3^	1.7 × 10^−3^	1.9 × 10^−3^	1.8 × 10^−3^
GOA	2-5-1	1.3 × 10^−2^	3.4 × 10^−2^	1.3 × 10^−2^	3.5 × 10^−2^
	2-10-1	1.2 × 10^−2^	4.0 × 10^−2^	1.2 × 10^−2^	4.0 × 10^−2^
	2-15-1	4.9 × 10^−3^	3.1 × 10^−3^	4.9 × 10^−3^	3.2 × 10^−3^
KHA	2-5-1	5.6 × 10^−3^	7.3 × 10^−3^	5.6 × 10^−3^	7.3 × 10^−3^
	2-10-1	6.9 × 10^−3^	8.7 × 10^−3^	7.0 × 10^−3^	9.0 × 10^−3^
	2-15-1	5.5 × 10^−3^	5.8 × 10^−3^	5.6 × 10^−3^	6.0 × 10^−3^
PSO	2-5-1	6.5 × 10^−3^	2.6 × 10^−3^	6.5 × 10^−3^	2.6 × 10^−3^
	2-10-1	9.0 × 10^−3^	3.0 × 10^−3^	9.1 × 10^−3^	3.1 × 10^−3^
	2-15-1	8.6 × 10^−3^	3.0 × 10^−3^	8.6 × 10^−3^	3.0 × 10^−3^
SHO	2-5-1	3.7 × 10^−3^	2.5 × 10^−3^	3.7 × 10^−3^	2.6 × 10^−3^
	2-10-1	3.5 × 10^−3^	2.1 × 10^−3^	3.6 × 10^−3^	2.1 × 10^−3^
	2-15-1	3.6 × 10^−3^	3.3 × 10^−3^	3.7 × 10^−3^	3.3 × 10^−3^
SSA	2-5-1	4.7 × 10^−3^	3.2 × 10^−3^	4.7 × 10^−3^	3.2 × 10^−3^
	2-10-1	4.1 × 10^−3^	2.3 × 10^−3^	4.1 × 10^−3^	2.2 × 10^−3^
	2-15-1	4.0 × 10^−3^	3.1 × 10^−3^	4.1 × 10^−3^	3.1 × 10^−3^
TSA	2-5-1	1.5 × 10^−2^	3.4 × 10^−2^	1.5 × 10^−2^	3.4 × 10^−2^
	2-10-1	2.5 × 10^−3^	1.6 × 10^−3^	2.6 × 10^−3^	1.7 × 10^−3^
	2-15-1	2.9 × 10^−3^	1.4 × 10^−3^	2.9 × 10^−3^	1.4 × 10^−3^
TSO	2-5-1	3.3 × 10^−3^	1.9 × 10^−3^	3.3 × 10^−3^	1.8 × 10^−3^
	2-10-1	2.7 × 10^−3^	1.5 × 10^−3^	2.8 × 10^−3^	1.5 × 10^−3^
	2-15-1	3.0 × 10^−3^	1.7 × 10^−3^	3.1 × 10^−3^	1.7 × 10^−3^

**Table 2:** Comparison of the results obtained for *n* = 20.

System	Network Structure	The Results			
		Train		Test	
		Mean	Std.	Mean	Std.
ABC	2-5-1	7.0 × 10^−3^	2.3 × 10^−3^	7.1 × 10^−3^	2.4 × 10^−3^
	2-10-1	8.9 × 10^−3^	8.2 × 10^−3^	8.9 × 10^−3^	8.5 × 10^−3^
	2-15-1	1.3 × 10^−2^	1.4 × 10^−2^	5.6 × 10^−2^	1.2 × 10^−1^
BOA	2-5-1	3.7 × 10^−2^	3.1 × 10^−2^	3.7 × 10^−2^	3.1 × 10^−2^
	2-10-1	2.1 × 10^−2^	1.8 × 10^−2^	2.2 × 10^−2^	1.8 × 10^−2^
	2-15-1	2.6 × 10^−2^	2.5 × 10^−2^	2.6 × 10^−2^	2.5 × 10^−2^
CS	2-5-1	5.5 × 10^−3^	1.6 × 10^−3^	5.6 × 10^−3^	1.6 × 10^−3^
	2-10-1	5.8 × 10^−3^	1.7 × 10^−3^	5.8 × 10^−3^	1.7 × 10^−3^
	2-15-1	6.5 × 10^−3^	2.1 × 10^−3^	6.6 × 10^−3^	2.2 × 10^−3^
CSO	2-5-1	3.9 × 10^−3^	2.3 × 10^−3^	4.0 × 10^−3^	2.4 × 10^−3^
	2-10-1	4.7 × 10^−3^	1.4 × 10^−3^	4.8 × 10^−3^	1.5 × 10^−3^
	2-15-1	6.9 × 10^−3^	2.3 × 10^−3^	7.1 × 10^−3^	2.5 × 10^−3^
DA	2-5-1	4.3 × 10^−3^	3.3 × 10^−3^	4.4 × 10^−3^	3.4 × 10^−3^
	2-10-1	4.0 × 10^−3^	3.2 × 10^−3^	4.1 × 10^−3^	3.3 × 10^−3^
	2-15-1	3.5 × 10^−3^	2.1 × 10^−3^	3.5 × 10^−3^	2.1 × 10^−3^
FA	2-5-1	5.0 × 10^−4^	3.9 × 10^−4^	5.2 × 10^−4^	4.0 × 10^−4^
	2-10-1	8.0 × 10^−4^	9.6 × 10^−4^	8.3 × 10^−4^	1.0 × 10^−3^
	2-15-1	5.4 × 10^−4^	4.6 × 10^−4^	5.4 × 10^−4^	4.7 × 10^−4^
GOA	2-5-1	3.5 × 10^−3^	2.3 × 10^−3^	3.6 × 10^−3^	2.4 × 10^−3^
	2-10-1	3.4 × 10^−3^	2.5 × 10^−3^	3.5 × 10^−3^	2.5 × 10^−3^
	2-15-1	3.7 × 10^−3^	3.1 × 10^−3^	3.7 × 10^−3^	3.1 × 10^−3^
KHA	2-5-1	1.5 × 10^−2^	1.2 × 10^−2^	1.5 × 10^−2^	1.2 × 10^−2^
	2-10-1	1.5 × 10^−2^	7.4 × 10^−3^	1.5 × 10^−2^	7.3 × 10^−3^
	2-15-1	1.9 × 10^−2^	1.4 × 10^−2^	1.9 × 10^−2^	1.4 × 10^−2^
PSO	2-5-1	1.0 × 10^−2^	4.4 × 10^−3^	1.0 × 10^−2^	4.5 × 10^−3^
	2-10-1	9.0 × 10^−3^	3.7 × 10^−3^	9.0 × 10^−3^	3.7 × 10^−3^
	2-15-1	9.4 × 10^−3^	3.2 × 10^−3^	9.5 × 10^−3^	3.2 × 10^−3^
SHO	2-5-1	2.7 × 10^−3^	1.7 × 10^−3^	2.8 × 10^−3^	1.7 × 10^−3^
	2-10-1	1.9 × 10^−3^	9.9 × 10^−4^	2.0 × 10^−3^	1.0 × 10^−3^
	2-15-1	1.6 × 10^−3^	6.3 × 10^−4^	1.6 × 10^−3^	6.5 × 10^−4^
SSA	2-5-1	5.0 × 10^−3^	2.9 × 10^−3^	5.0 × 10^−3^	3.0 × 10^−3^
	2-10-1	5.6 × 10^−3^	3.6 × 10^−3^	5.7 × 10^−3^	3.7 × 10^−3^
	2-15-1	4.4 × 10^−3^	2.7 × 10^−3^	4.5 × 10^−3^	2.7 × 10^−3^
TSA	2-5-1	5.1 × 10^−3^	8.5 × 10^−3^	5.1 × 10^−3^	7.9 × 10^−3^
	2-10-1	2.5 × 10^−3^	1.3 × 10^−3^	2.6 × 10^−3^	1.3 × 10^−3^
	2-15-1	2.9 × 10^−3^	1.5 × 10^−3^	3.0 × 10^−3^	1.9 × 10^−3^
TSO	2-5-1	3.6 × 10^−3^	2.4 × 10^−3^	3.6 × 10^−3^	2.3 × 10^−3^
	2-10-1	3.2 × 10^−3^	1.8 × 10^−3^	3.3 × 10^−3^	1.8 × 10^−3^
	2-15-1	3.3 × 10^−3^	1.5 × 10^−3^	3.4 × 10^−3^	1.6 × 10^−3^

**Table 3:** Comparison of the results obtained for *n* = 50.

System	Network Structure	The Results			
		Train		Test	
		Mean	Std.	Mean	Std.
ABC	2-5-1	1.2 × 10^−2^	1.0 × 10^−2^	1.3 × 10^−2^	1.0 × 10^−2^
	2-10-1	1.1 × 10^−2^	5.0 × 10^−3^	1.1 × 10^−2^	5.0 × 10^−3^
	2-15-1	1.3 × 10^−2^	6.2 × 10^−3^	1.3 × 10^−2^	6.0 × 10^−3^
BOA	2-5-1	2.5 × 10^−2^	1.6 × 10^−2^	2.5 × 10^−2^	1.6 × 10^−2^
	2-10-1	1.5 × 10^−2^	1.2 × 10^−2^	1.5 × 10^−2^	1.2 × 10^−2^
	2-15-1	1.8 × 10^−2^	1.2 × 10^−2^	1.8 × 10^−2^	1.2 × 10^−2^
CS	2-5-1	8.2 × 10^−3^	2.3 × 10^−3^	8.3 × 10^−3^	2.3 × 10^−3^
	2-10-1	7.5 × 10^−3^	2.4 × 10^−3^	7.6 × 10^−3^	2.4 × 10^−3^
	2-15-1	8.1 × 10^−3^	3.0 × 10^−3^	8.2 × 10^−3^	2.9 × 10^−3^
CSO	2-5-1	7.3 × 10^−3^	2.9 × 10^−3^	7.3 × 10^−3^	2.9 × 10^−3^
	2-10-1	7.0 × 10^−3^	2.6 × 10^−3^	7.1 × 10^−3^	2.6 × 10^−3^
	2-15-1	7.6 × 10^−3^	2.8 × 10^−3^	7.8 × 10^−3^	2.8 × 10^−3^
DA	2-5-1	3.8 × 10^−3^	3.0 × 10^−3^	3.9 × 10^−3^	3.0 × 10^−3^
	2-10-1	4.6 × 10^−3^	3.5 × 10^−3^	4.7 × 10^−3^	3.5 × 10^−3^
	2-15-1	4.1 × 10^−3^	2.5 × 10^−3^	4.2 × 10^−3^	2.6 × 10^−3^
FA	2-5-1	4.5 × 10^−4^	2.3 × 10^−4^	4.6 × 10^−4^	2.3 × 10^−4^
	2-10-1	5.5 × 10^−4^	2.8 × 10^−4^	5.7 × 10^−4^	2.8 × 10^−4^
	2-15-1	8.6 × 10^−4^	5.5 × 10^−4^	8.9 × 10^−4^	5.8 × 10^−4^
GOA	2-5-1	2.7 × 10^−3^	1.9 × 10^−3^	2.8 × 10^−3^	1.9 × 10^−3^
	2-10-1	2.3 × 10^−3^	1.6 × 10^−3^	2.4 × 10^−3^	1.7 × 10^−3^
	2-15-1	3.3 × 10^−3^	2.2 × 10^−3^	3.4 × 10^−3^	2.3 × 10^−3^
KHA	2-5-1	1.6 × 10^−2^	6.9 × 10^−3^	1.6 × 10^−2^	6.8 × 10^−3^
	2-10-1	1.8 × 10^−2^	7.2 × 10^−3^	1.8 × 10^−2^	7.2 × 10^−3^
	2-15-1	1.6 × 10^−2^	5.6 × 10^−3^	1.6 × 10^−2^	5.6 × 10^−3^
PSO	2-5-1	1.1 × 10^−2^	4.0 × 10^−3^	1.1 × 10^−2^	4.1 × 10^−3^
	2-10-1	1.0 × 10^−2^	3.9 × 10^−3^	1.0 × 10^−2^	3.9 × 10^−3^
	2-15-1	1.1 × 10^−2^	3.8 × 10^−3^	1.1 × 10^−2^	3.7 × 10^−3^
SHO	2-5-1	2.3 × 10^−3^	1.4 × 10^−3^	2.3 × 10^−3^	1.5 × 10^−3^
	2-10-1	1.9 × 10^−3^	8.4 × 10^−4^	2.0 × 10^−3^	8.9 × 10^−4^
	2-15-1	3.3 × 10^−3^	4.2 × 10^−3^	3.3 × 10^−3^	4.1 × 10^−3^
SSA	2-5-1	7.4 × 10^−3^	3.8 × 10^−3^	7.6 × 10^−3^	3.8 × 10^−3^
	2-10-1	5.3 × 10^−3^	2.8 × 10^−3^	5.4 × 10^−3^	2.8 × 10^−3^
	2-15-1	5.7 × 10^−3^	3.8 × 10^−3^	5.8 × 10^−3^	3.9 × 10^−3^
TSA	2-5-1	3.7 × 10^−3^	2.6 × 10^−3^	3.7 × 10^−3^	2.6 × 10^−3^
	2-10-1	3.4 × 10^−3^	2.3 × 10^−3^	3.5 × 10^−3^	2.3 × 10^−3^
	2-15-1	3.1 × 10^−3^	1.3 × 10^−3^	3.2 × 10^−3^	1.4 × 10^−3^
TSO	2-5-1	4.1 × 10^−3^	2.5 × 10^−3^	4.1 × 10^−3^	2.6 × 10^−3^
	2-10-1	5.3 × 10^−3^	2.8 × 10^−3^	5.3 × 10^−3^	2.8 × 10^−3^
	2-15-1	5.7 × 10^−3^	2.6 × 10^−3^	5.9 × 10^−3^	2.6 × 10^−3^

**Table 4:** Information on the best train mean error values obtained using related metaheuristic algorithms.

Algorithm	Train			
	Network Structure	Population Size	Mean	Std.
ABC	2-5-1	10	5.0 × 10^−3^	2.4 × 10^−3^
BOA	2-10-1	50	1.5 × 10^−2^	1.2 × 10^−2^
CS	2-5-1	10	3.9 × 10^−3^	1.1 × 10^−3^
CSO	2-10-1	10	3.7 × 10^−3^	2.6 × 10^−3^
DA	2-15-1	20	3.5 × 10^−3^	2.1 × 10^−3^
FA	2-5-1	50	4.5 × 10^−4^	2.3 × 10^−4^
GOA	2-10-1	50	2.3 × 10^−3^	1.6 × 10^−3^
KHA	2-15-1	10	5.5 × 10^−3^	5.8 × 10^−3^
PSO	2-5-1	10	6.5 × 10^−3^	2.6 × 10^−3^
SHO	2-15-1	20	1.6 × 10^−3^	6.3 × 10^−4^
SSA	2-15-1	10	4.0 × 10^−3^	3.1 × 10^−3^
TSA	2-10-1	10	2.5 × 10^−3^	1.6 × 10^−3^
TSO	2-10-1	10	2.7 × 10^−3^	1.5 × 10^−3^

**Table 5:** Information on the best test mean error values obtained using related metaheuristic algorithms.

Algorithm	Test			
	Network Structure	Population Size	Mean	Std.
ABC	2-5-1	10	5.1 × 10^−3^	2.4 × 10^−3^
BOA	2-10-1	50	1.5 × 10^−2^	1.2 × 10^−2^
CS	2-5-1	10	3.9 × 10^−3^	1.1 × 10^−3^
CSO	2-10-1	10	3.8 × 10^−3^	2.6 × 10^−3^
DA	2-15-1	20	3.5 × 10^−3^	2.1 × 10^−3^
FA	2-5-1	50	4.6 × 10^−4^	2.3 × 10^−4^
GOA	2-10-1	50	2.4 × 10^−3^	1.7 × 10^−3^
KHA	2-15-1	10	5.6 × 10^−3^	6.0 × 10^−3^
PSO	2-5-1	10	6.5 × 10^−3^	2.6 × 10^−3^
SHO	2-15-1	20	1.6 × 10^−3^	6.5 × 10^−4^
SSA	2-15-1	10	4.1 × 10^−3^	3.1 × 10^−3^
TSA	2-10-1	10	2.6 × 10^−3^	1.7 × 10^−3^
TSO	2-10-1	10	2.8 × 10^−3^	1.5 × 10^−3^

**Table 6:** General success scores according to the best results of related metaheuristic algorithms.

Order	Algorithm	Train Ranking Score	Test Ranking Score	Total Score
1	FA	1	1	2
2	SHO	2	2	4
3	GOA	3	3	6
4	TSA	4	4	8
5	TSO	5	5	10
6	DA	6	6	12
7	CSO	7	7	14
8	CS	8	9	17
9	SSA	9	8	17
10	ABC	10	10	20
11	KHA	11	11	22
12	PSO	12	12	24
13	BOA	13	13	26

## Data Availability

Not applicable.

## Abbreviations

The following abbreviations are used in this manuscript:

BOA	Butterfly optimization algorithm
SSA	Salp swarm algorithm
FA	Firefly algorithm
ABC	Artificial bee colony
PSO	Particle swarm optimization
KHA	Krill herd algorithm
CS	Cuckoo search
FFNN	Feed-forward neural network
ANN	Artificial neural network
GOA	Grasshopper optimization algorithm
ANFIS	Adaptive Network Fuzzy Inference System
DA	Dragonfly algorithm
SHO	Selfish herd optimizer
TSA	Tunicate swarm algorithm
TSO	Tuna swarm optimization
CSO	Chicken swarm optimization
MPP	Maximum power point
MPPT	Maximum power point tracking
PV	Photovoltaic
P&O	Perturb and observe
INC	Incremental Conductance
PID	Proportional Integral Derivative
FLC	Fuzzy Logic Control
PS	Photovoltaic System
SGO	Social group optimization
SMO	Slime mould optimization
RNNs	Recurrent neural networks
ACO	Ant colony optimization
RBFN	Radial basis function network
